# Zero-to-Two Nanoarchitectonics: Fabrication of Two-Dimensional Materials from Zero-Dimensional Fullerene

**DOI:** 10.3390/molecules26154636

**Published:** 2021-07-30

**Authors:** Guoping Chen, Lok Kumar Shrestha, Katsuhiko Ariga

**Affiliations:** 1 Graduate School of Frontier Sciences, The University of Tokyo, 5-1-5 Kashiwanoha, Kashiwa, Chiba 277-8561, Japan; 9290561136@edu.k.u-tokyo.ac.jp; 2 WPI Research Center for Materials Nanoarchitectonics (MANA), National Institute for Materials Science (NIMS), 1-1 Namiki, Ibaraki, Tsukuba 305-0044, Japan; SHRESTHA.Lokkumar@nims.go.jp

**Keywords:** fullerene, interface, nanoarchitectonics, nanosheet, self-assembly, two-dimensional material

## Abstract

Nanoarchitectonics of two-dimensional materials from zero-dimensional fullerenes is mainly introduced in this short review. Fullerenes are simple objects with mono-elemental (carbon) composition and zero-dimensional structure. However, fullerenes and their derivatives can create various types of two-dimensional materials. The exemplified approaches demonstrated fabrications of various two-dimensional materials including size-tunable hexagonal fullerene nanosheet, two-dimensional fullerene nano-mesh, van der Waals two-dimensional fullerene solid, fullerene/ferrocene hybrid hexagonal nanosheet, fullerene/cobalt porphyrin hybrid nanosheet, two-dimensional fullerene array in the supramolecular template, two-dimensional van der Waals supramolecular framework, supramolecular fullerene liquid crystal, frustrated layered self-assembly from two-dimensional nanosheet, and hierarchical zero-to-one-to-two dimensional fullerene assembly for cell culture.

## 1. Introduction

As a game-changer of science and technology in the late 20th to 21st century, nanotechnology has great contributions in explorations on nanoscale materials and their phenomena. Developments in nanotechnology-based observation/evaluation techniques [1,2,3] and manipulation methods even in atom/molecular scales [4,5,6] open ways to control nanoscale structures and create nano-specific functions such as highly anisotropic electronic and optical properties. These research trends make us realize the importance of nanoscale structures in material functions. In parallel, basic areas and emerging fields for material production have continuously progressed. Functional materials in huge varieties have been prepared and fabricated through efforts in organic synthesis [7,8,9,10], polymer chemistry [11,12,13], coordination chemistry [14,15,16,17], supramolecular chemistry [18,19,20,21], and the other materials science especially for nanostructured materials [22,23,24,25]. Various fabrication techniques including the self-assembled monolayer (SAM) method [26,27,28], Langmuir–Blodgett (LB) technique [29,30], and layer-by-layer (LbL) assembly [31,32] have indispensable roles in the developments of functional materials as well as contributions with emerging materials such as nanocarbon materials [33,34] and nanoporous materials [35,36].

On the basis of these developments of science and technology, various functional materials and systems have been continuously produced upon important social demands in energy [37,38,39,40], environmental [41,42,43,44], and biomedical fields [45,46,47,48]. It becomes realized that regulation of nanostructures within materials systems is crucially important for getting better performances with higher efficiencies. Interactions in well-coordinated components leads to efficient processes in many events including conductivity, reactivity, adhesion, and optical response. Both materials’ intrinsic qualities and their precise internal structures are indispensable factors to produce more desirable materials for target functions. Therefore, knowledge and techniques in nanotechnology and the other research fields have to be combined into a unified new paradigm. This task is assigned to an emerging concept, nanoarchitectonics [49].

**Figure 1:**
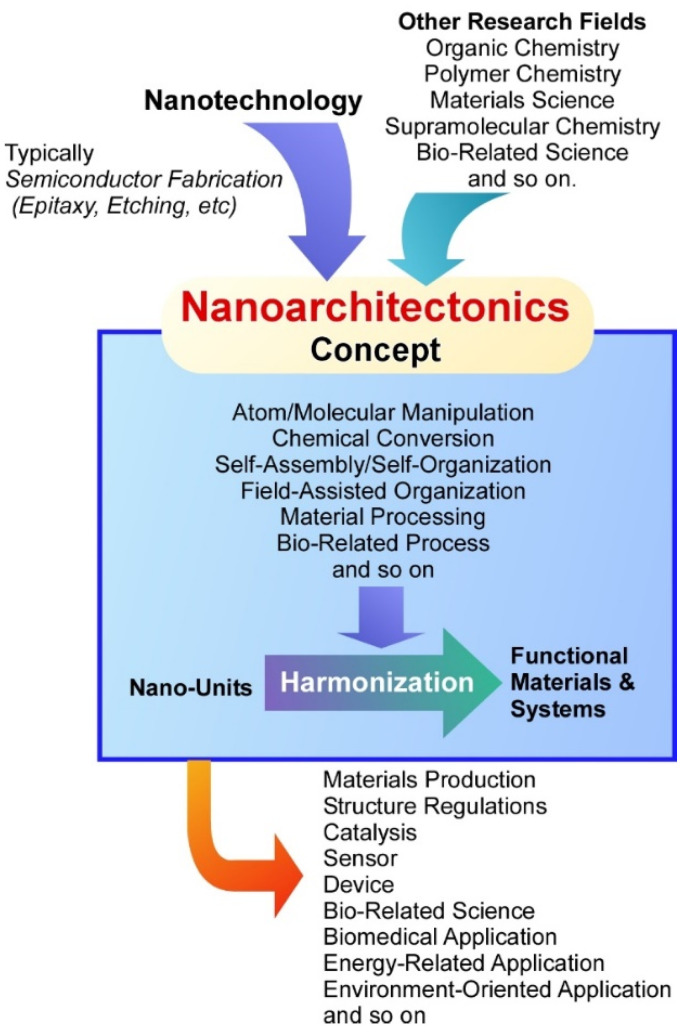
Outline of the nanoarchitectonics concept and contributing fields.

As the nanotechnology concept is said to be initiated by a lecture by Richard Feynman [50,51], nanoarchitectonics concept in this meaning was first proposed by Masakazu Aono [52,53] at the conference held in 2000. Nanoarchitectonics unifies nanotechnology with other research fields such as organic chemistry, polymer chemistry, materials science, supramolecular chemistry, and bio-related science [54] (Figure 1). Based on the nanoarchitectonics strategies, functional material systems can be produced from nanoscale unit components through the combinations and selections of various effects and the processes including atom/molecular manipulation, chemical conversion, self-assembly/self-organization, field-assisted organization, material processing, and bio-related process [55]. Because molecular and material interactions at the nanoscale cannot ignore the influences of various ambiguities such as thermal fluctuations, statistical distributions, and quantum effects, nanoarchitectonics fabrications are often based on the harmonization of unit processes rather than their simple summation [56]. In many cases of functional materials production based on nanoarchitectonics procedures, several processes are used together and sequential assembly and fabrications are applied. Therefore, the nanoarchitectonics processes are advantageous to produce hierarchical and asymmetrical structures rather than equilibrated self-assembly often used in supramolecular chemistry [57]. These features can be commonly applied to a wide range of materials. Therefore, the nanoarchitectonics concept has been applied in various research fields including materials production [58,59], structure regulations [60,61], catalysis [62,63], sensor [64,65], device [66,67], bio-related science [68,69,70], biomedical applications [71,72], energy-related applications [73,74], and environment-oriented applications [75,76].

As generally known dimensionality of materials is a crucial factor to determine functions and properties of the materials. In addition to zero-dimensional [77,78,79], one-dimensional [80,81,82], three-dimensional [83,84,85], and further integrated functional materials [86,87], various two-dimensional materials have been paid special attention [88,89,90]. Two-dimensional materials have their unique electronic properties [91,92] and play important roles in interfacial sciences [93,94]. As typically seen in graphene as a representative two-dimensional material, many attractive properties such as optical transparency, mechanical strength, thermal conductivity, and carrier mobility are highly expected. These features can be potentially used for device applications. Unlike zero-dimensional and one-dimensional materials, two-dimensional materials can efficiently form interfacial environments that are useful as interface for devices such as sensors. The method of synthesis has strong influence on these properties. Therefore, two-dimensional materials are selected as subjects of nanoarchitectonics research in this review article.

**Figure 2:**
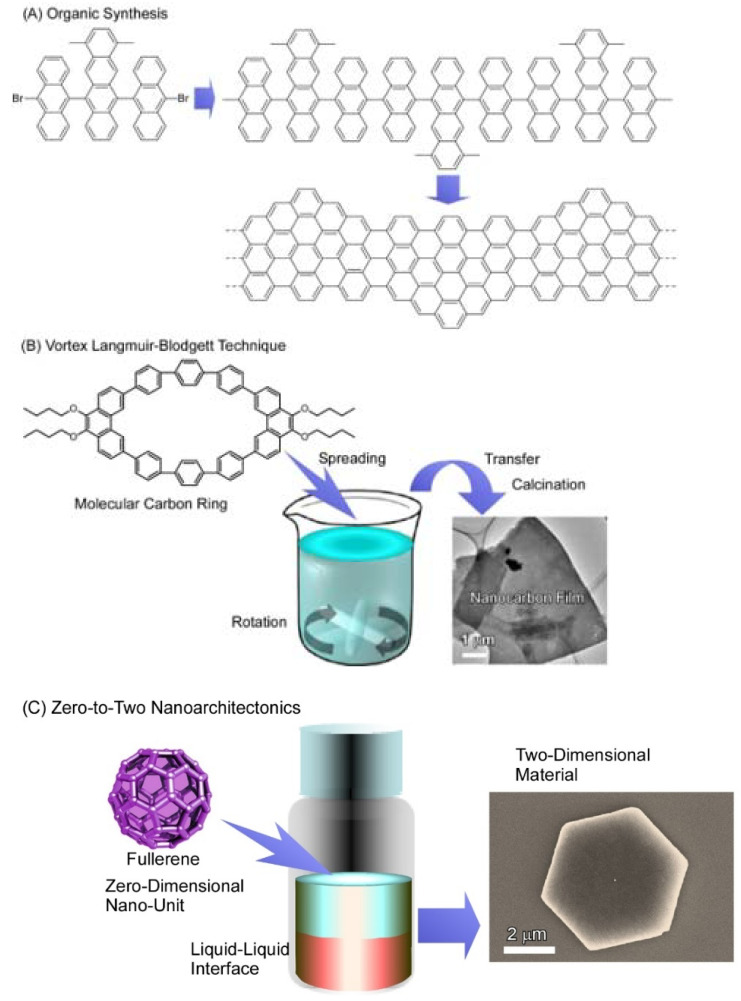
Emerging methods for the fabrication of two-dimensional carbon materials; (**A**) organic synthesis; (**B**) vortex Langmuir–Blodgett technique; (**C**) zero-to-two nanoarchitectonics (through liquid–liquid interfacial precipitation method).

Two-dimensional materials can be fabricated with some physical methods such as exfoliation [95,96] and chemical vapor deposition [97,98] as standard procedures. Some bottom-up-type fabrications of two-dimensional materials have been also explored as illustrated in Figure 2. For example, syntheses of graphene-like two-dimensional nanocarbon pieces such as two-dimensional graphene nanoribbon upon well-designed organic synthesis have been also demonstrated (Figure 2A) [99,100]. Preparation of two-dimensional nanocarbon films from molecular carbon rings was realized using a novel technique, vortex Langmuir–Blodgett method, and subsequent calcination procedure (Figure 2B) [101]. Among bottom-up-type fabrications, fabrications of two-dimensional structures and assemblies from zero-dimensional fullerenes are mainly introduced in this short review article (Figure 2C). Fullerenes are very simple objects with mono-elemental (carbon) composition and zero-dimensional structure. As demonstrated in the following sections, simple unit components of fullerenes and their derivatives can create various types of two-dimensional materials. In this review article, essences of zero-to-two nanoarchitectonics for the fabrication of two-dimensional materials from zero-dimensional fullerene molecules are discussed.

## 2. Two-Dimensional Fullerene Nanoarchitecture

For the preparation of self-assembled fullerene nanocrystals, a simple liquid–liquid interface precipitation method is often used [102,103]. In the liquid–liquid interface precipitation method, the crystal formation mechanism is supposed to be driven by supersaturation of fullerene molecules at interfaces between good solvent (with higher fullerene solubility) and bad solvent (with lower fullerene solubility). Although the preparation procedure of this technique is very simple, fullerene assemblies in various dimensions—including one-dimensional rods/tubes [104,105], three-dimensional cubes [106,107], and their hierarchical nanoarchitectures [108,109]—can be obtained. For example, Sathish and Miyazawa synthesized size-tunable C_60_ hexagonal thin crystalline nanosheets through the liquid–liquid interface precipitation method at the carbon tetrachloride (CCl_4_)/alcohol interface for the first time [110]. The size of the hexagonal nanosheets could be tailored merely by selecting proper alcohol for the interfacial precipitation. Hexagonal nanosheet with ~7.5 μm diameter was observed for the CCl_4_/isopropyl alcohol interface, whereas the hexagonal nanosheets with ~2.5 μm diameter and 500 nm diameter at the CCl_4_/ethanol and CCl_4_/methanol interface, respectively. This work opens a new door for synthesizing fullerene nanosheets by simple self-assembly.

**Figure 3:**
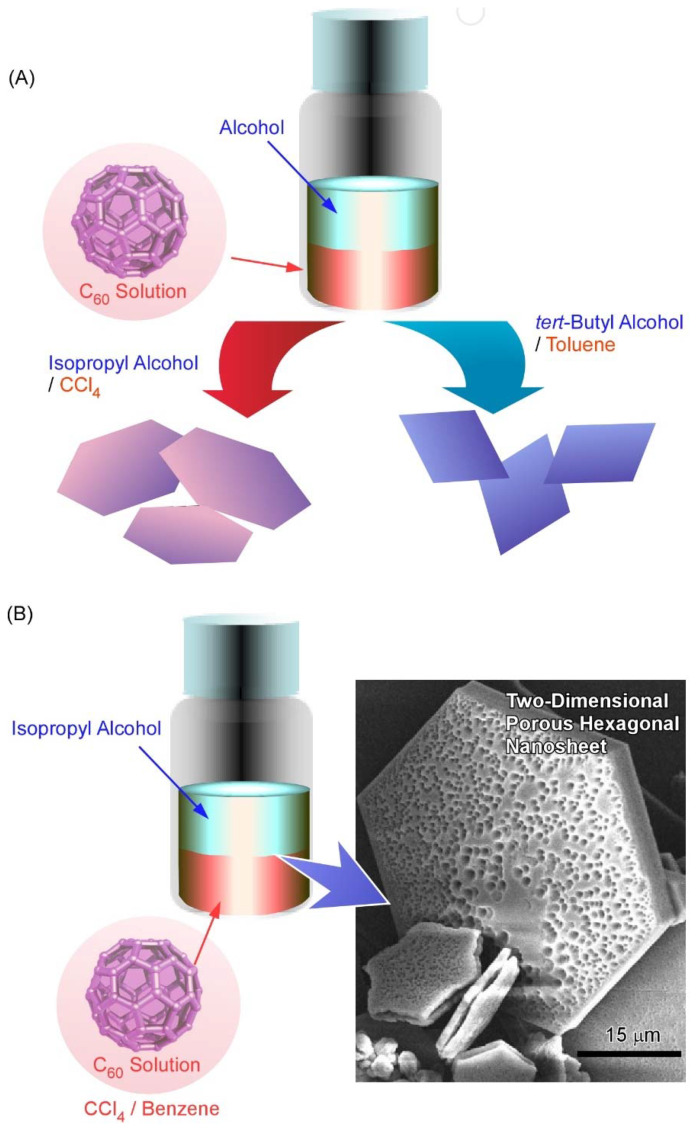
(**A**) Fabrication of variously shaped two-dimensional fullerene assemblies through appropriate choice of solvent combinations for the liquid–liquid interfacial precipitation method. (**B**) Two-dimensional nanosheets possessing dimorphic macroporous/mesoporous structures fabricated via C_60_ assembly at interfaces between isopropyl alcohol and CCl_4_/benzene mixtures.

It was demonstrated in further studies that variations in the shape and size of two-dimensional fullerene assemblies can be fabricated with the appropriate choice of solvent combinations for the liquid–liquid interfacial precipitation method (Figure 3A) [111]. Formation of hexagonal C_60_ nanosheets was achieved at the interfaces between toluene and isopropyl alcohol, while two-dimensional nanosheets with uniform rhombi and hexagonal shapes were obtained through the self-assembly of C_60_ molecules at the interfaces of toluene/*tert*-butyl alcohol and CCl_4_/isopropyl alcohol, respectively. The lattice formation favoring these two-dimensional crystalline assemblies may be the most influential factor in determining the shape of the two-dimensional nanosheets. Under certain stimuli, such as contact with water, two-dimensional rhombi-shaped nanosheets were transformed into one-dimensional nanorods. The latter transformation would be occurred accompanied by the release of high surface energy.

Pore formation within a two-dimensional C_60_ nanosheet was achieved by faint adaptations of solvent systems to the liquid–liquid interfacial precipitation system [112]. Two-dimensional nanosheets possessing dimorphic macroporous/interporous structures were fabricated via C_60_ assembly at interfaces between isopropyl alcohol and CCl_4_/benzene mixtures (Figure 3B). At high CCl_4_ contents, macropores were formed in the two-dimensional hexagonal nanosheets. In addition, smaller mesopores were also formed within the macropores under suitable mixing conditions between CCl_4_ and benzene. The average mesopore sizes came to be 15 to 25 nm and 20 to 40 nm at CCl_4_/benzene mixing ratios of 80:20 and 90:10, respectively. The latter two-dimensional structures with bimodal features were accompanied by mixed *fcc* and *hcp* crystal structures. It is expected that the evaporation of solvents trapped in them would be the cause of mesopore formation. The formed bimodal nanoarchitectures have high surface areas with enhanced electrochemical activities, which would be useful for energy-related applications.

**Figure 4:**
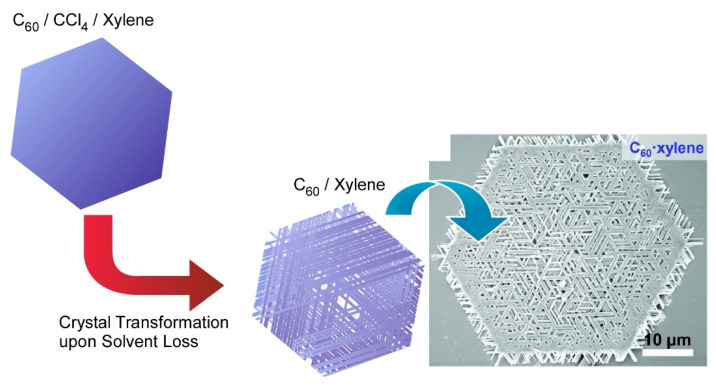
Crystal transformation strategy to transform two-dimensional C_60_ microplates to nano-meshes made of ordered nanorods. Reprinted with permission from [113]. Copyright 2019 Royal Society of Chemistry.

Chen and co-workers developed a simple but effective co-solvent induced crystal transformation strategy to transform two-dimensional C_60_ microplates to nano-meshes made of ordered nanorods (Figure 4) [113]. The transformation was triggered by removing the solvent molecule and then re-incorporating another solvent molecule. Specifically, C_60_ plates were obtained first by adding isopropyl alcohol to the CCl_4_/*m*-xylene mixture with gentle swirling. The C_60_ plates were concentrated by centrifugation and then diluted with isopropyl alcohol to transform to two-dimensional mesh networks. After diluting with isopropyl alcohol, the incorporated CCl_4_/*m*-xylene would then leave the C_60_ plate, leading to the formation of pores in the C_60_ microplates. Under appropriate conditions, *m*-xylene can be re-incorporated during the C_60_ recrystallization and eventually gives rise to the mesh network. This work opens a new field for designing hierarchical two-dimensional superstructures. The fabricated complicated two-dimensional mesh-like fullerene superstructures would be useful separation and filtration such as oil/water separation and biomolecular segregations.

## 3. Two-Dimensional Nanoarchitecture from Fullerene with Other Component

**Figure 5:**
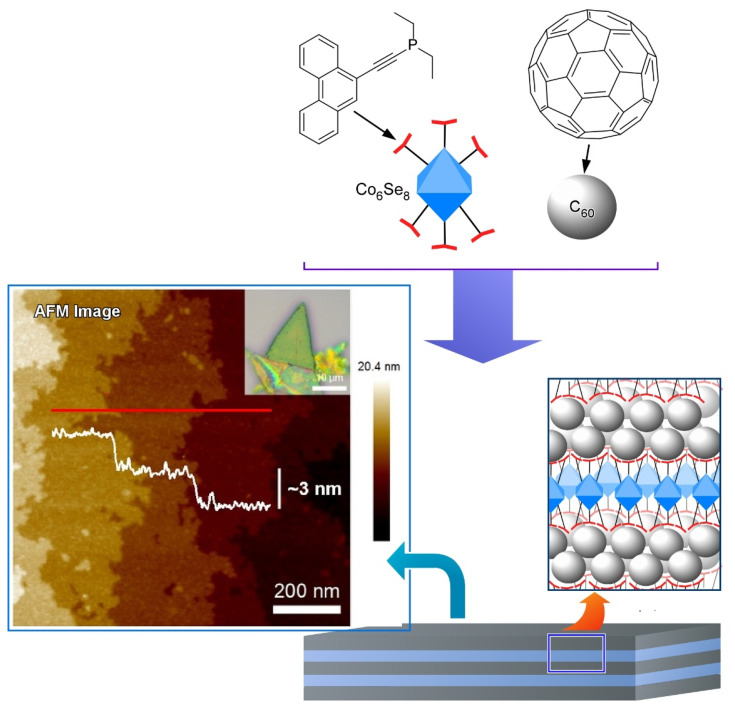
Two-dimensionally confined fullerene as strippable layer of van der Waals solid from a structure-directing building block (Co_6_Se_8_-based cluster) and C_60_ fullerene by self-assembly method. Reprinted with permission from [114]. Copyright 2016 American Chemical Society.

Steigerwald, Nuckolls, Roy, and coworkers proposed a method that utilizes dimensionally confined fullerene as a strippable layer to obtain two-dimensional fullerene (Figure 5) [114]. They firstly synthesized a layered van der Waals solid from a structure-directing building block and C_60_ fullerene by self-assembly method. Transition metal chalcogenide cluster (Co_6_Se_8_(PEt_2_phen)_6_) was employed as the structure-directing building block that associated with C_60_ and thereby directed the spontaneous assembly of C_60_ into crystalline solid of [Co_6_Se_8_(PEt_2_phen)_6_][C_60_]_5_. The structure of [Co_6_Se_8_(PEt_2_phen)_6_][C_60_]_5_ is composed of two layers stacked along the hexagonal *c*-axis in which a slightly corrugated trigonal array of C_60_ was separated by a layer comprised of a molecular cluster with stoichiometry [Co_6_Se_8_(PEt_2_phen)_6_][C_60_]_2_. The intralayer bonding of [Co_6_Se_8_(PEt_2_phen)_6_][C_60_]_5_ is strong enough while interlayer bonding is weak enough that the layered solid may be mechanically exfoliated. Therefore, they exfoliated the layered crystals to produce two-dimensional sheets as thin as ~130 nm with smooth surfaces and transferred them onto Si wafers. In this example, flexible molecular recognition between fullerene and ligand units enables us to nanoarchitect van der Waals materials from multiple components in desired layered structures.

**Figure 6:**
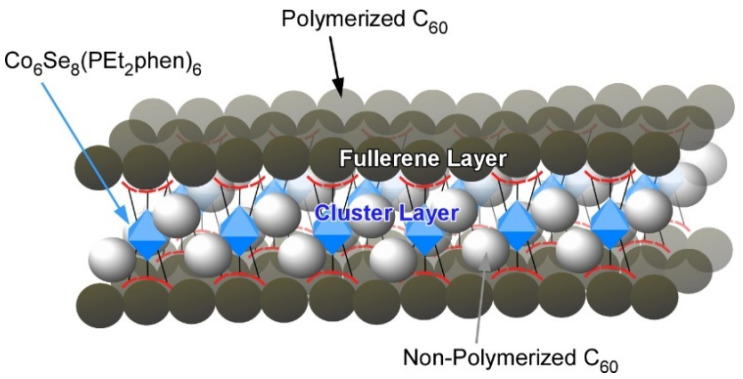
Isolated free-standing two-dimensional crystalline C_60_ structure fabricated by photopolymerization and solvent-induced exfoliation.

Based on the above-mentioned work, Zhu, Roy, and coworkers made an effort to enhance the intralayer interactions of the layered van der Waals solid [Co_6_Se_8_(PEt_2_phen)_6_][C_60_]_5_ by photo converting method and isolated free-standing two-dimensional crystalline C_60_ structure by solvent-induced exfoliation (Figure 6) [115]. Specifically, they first used a 532 nm laser to polymerize [Co_6_Se_8_(PEt_2_phen)_6_][C_60_]_5_ for 5–10 days to convert the layer into a covalently linked sheet of polymerized C_60_. The irradiated crystals of [Co_6_Se_8_(PEt_2_phen)_6_][C_60_]_5_ readily dissolve in toluene to produce a brown solution with a dark brown suspension of polymerized C_60_ sheets. The two-dimensional polymerized C_60_ sheets were composed of a layer of the inorganic cluster, sandwiched between two monolayers of polymerized C_60_. The nanosheets with 5 nm thick can be transferred onto solid substrates and depolymerized by heating.

**Figure 7:**
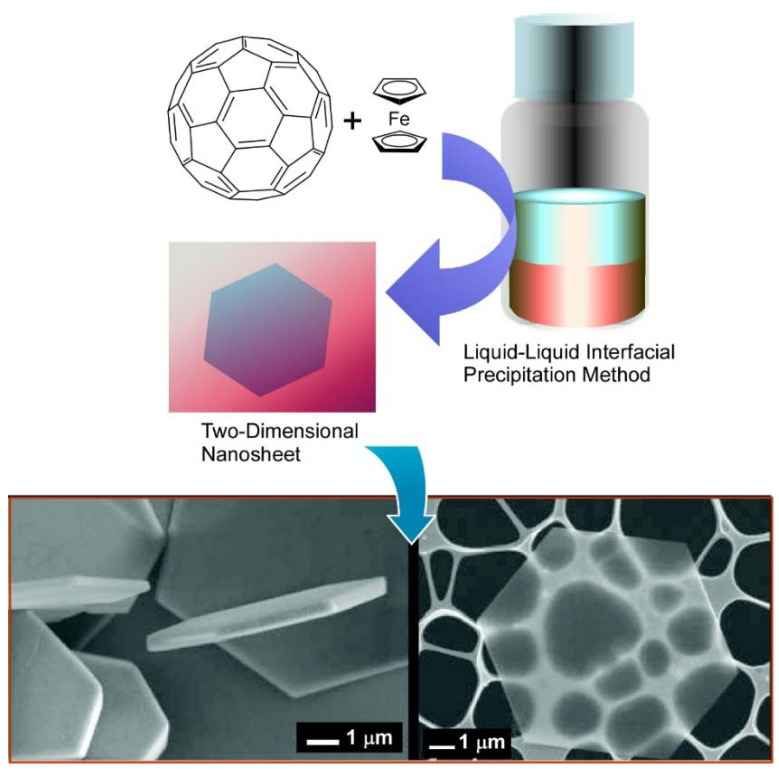
Two-dimensional C_60_/ferrocene nanosheets prepared by liquid–liquid interfacial precipitation method. Reprinted with permission from [116]. Copyright 2009 American Chemical Society.

Wakahara, Tateyama, and coworkers prepared C_60_/ferrocene nanosheets by a simple liquid–liquid interfacial precipitation method through the formation of an interface between isopropyl alcohol and toluene solution containing C_60_ and ferrocene (Figure 7) [116]. A strong charge-transfer band of diffuse reflectance spectra between ferrocene and C_60_ was observed at 782 nm, indicating the presence of donor−acceptor interaction in the nanosheets. The average size of the C_60_/ ferrocene hexagonal nanosheets was 9.1 ± 6.2 μm and the thickness is about 250−550 nm. Size would be controlled by conditions at the liquid–liquid interfacial precipitation method. In the case of C_60_/ferrocene nanosheets, each C_60_ is surrounded by two ferrocene molecules, showing a triclinic C_60_(ferrocene)_2_ structure. Upon heating the nanosheets to 150 °C to sublimate ferrocene, the C_60_/ferrocene hexagonal nanosheets can be converted to *fcc* C_60_ hexagonal nanosheets with the same shape and size as the C_60_/ferrocene nanosheets. The successful preparation of C_60_/ferrocene nanosheets based on the co-crystallization in the liquid–liquid interface is expected to be an important steppingstone to the fabrication of novel fullerene nanosheets and to lead to a new field relative to fullerene-derived co-crystals engineering.

In addition to synthesizing C_60_/ferrocene hexagonal nanosheets co-crystals nanosheets, Wakahara, Bradley, Anthopoulos, and coworkers also reported the preparation of novel co-crystals nanosheets comprising C_60_ and 5,10,15,20-tetrakis(4-methoxyphenyl) porphyrinato cobalt(II) (CoTMPP) using liquid–liquid interfacial precipitation method [117]. The hybrid nanosheets were formed at an interface between isopropyl alcohol and toluene solution containing C_60_/CoTMPP. The thickness of the nanosheets is ~50–200 nm. The C_60_/CoTMPP co-crystals nanosheets exhibited ambipolar transport characteristics with nearly balanced hole/electron mobilities. The successful preparation of fullerene-derived co-crystals nanosheets by using the liquid–liquid interfacial precipitation method to co-crystallization would enrich the fabrication of two-dimensional fullerene.

**Figure 8:**
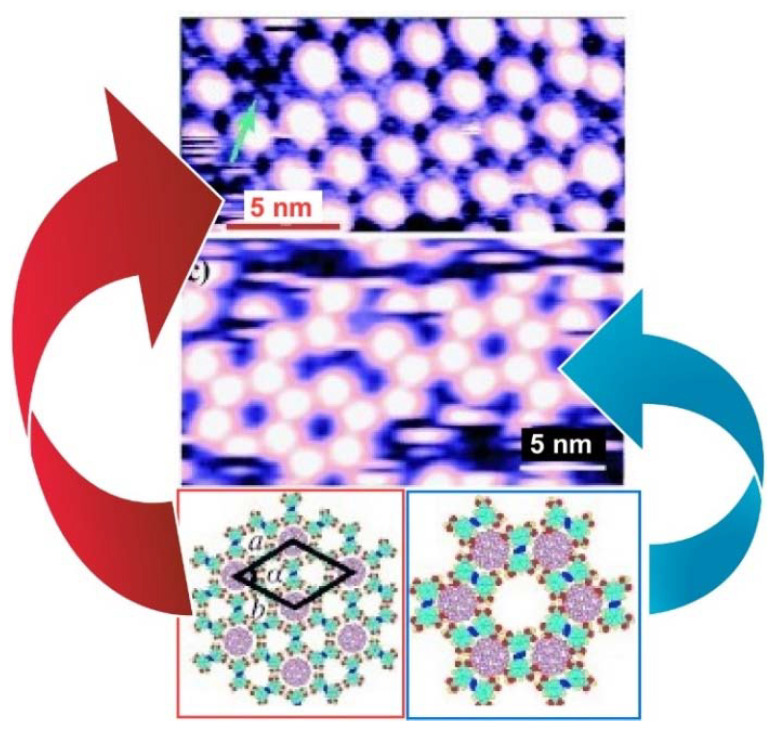
Two-dimensional C_60_ arrangement on two types of cavities formed by an azobenzene derivative (NN4A). Reprinted with permission from [118]. Copyright 2009 Wiley-VCH.

Zeng, Wang, and coworkers reported a two-dimensional molecular template with two types of cavities that have different sizes and symmetry at the liquid–solid interface formed by an azobenzene derivative (NN4A) [118] (Figure 8). Both the A- (diameter: 12.0 Å) and B-type (diameter: 8.6 Å) cavities were large enough in diameter to accommodate guest molecules such as fullerene molecules. Therefore, highly ordered fullerene arrays were constructed at the liquid–solid interface of graphite by coadsorption of fullerenes and NN4A. The experimental and theoretical results indicate that C_60_ molecules (diameter:6.80 Å) occupy both types of the cavity, while the larger C_80_ (diameter: 8.22 Å) and Sc_3_N@C_80_ molecules were trapped exclusively in the A-type cavities, which implies appreciable site selectivity. These template-directed fabrications of fullerene arrays provide a new method for the formation of two-dimensional fullerene.

Gao, Pan, Guo, and coworkers investigated a two-dimensional van der Waals supramolecular framework consisting of C_60_ molecules and octane thiol molecules on the Au(111) substrate by self-assembled co-crystallization method [119]. The porous framework was a result of cooperative self-organization with the C_60_ molecule and octane thiol as cohosts of the framework, in which the pores were filled by RS-Au-SR (R = CH_3_(CH_2_)_7_S) staples. The pairing of two adjacent C_60_ molecules and the formation of the C_60_ dimer were visualized, showing a charge transfer in each C_60_ dimer. The presence of octane thiol molecules prevents C_60_ from forming the preferred close-packed structure. The shapes of grown frameworks should be mainly influenced by the van der Waals interactions between the alkane chain and the C_60_ molecules.

## 4. Two-Dimensional Nanoarchitecture from Alkylated Fullerene Derivative

**Figure 9:**
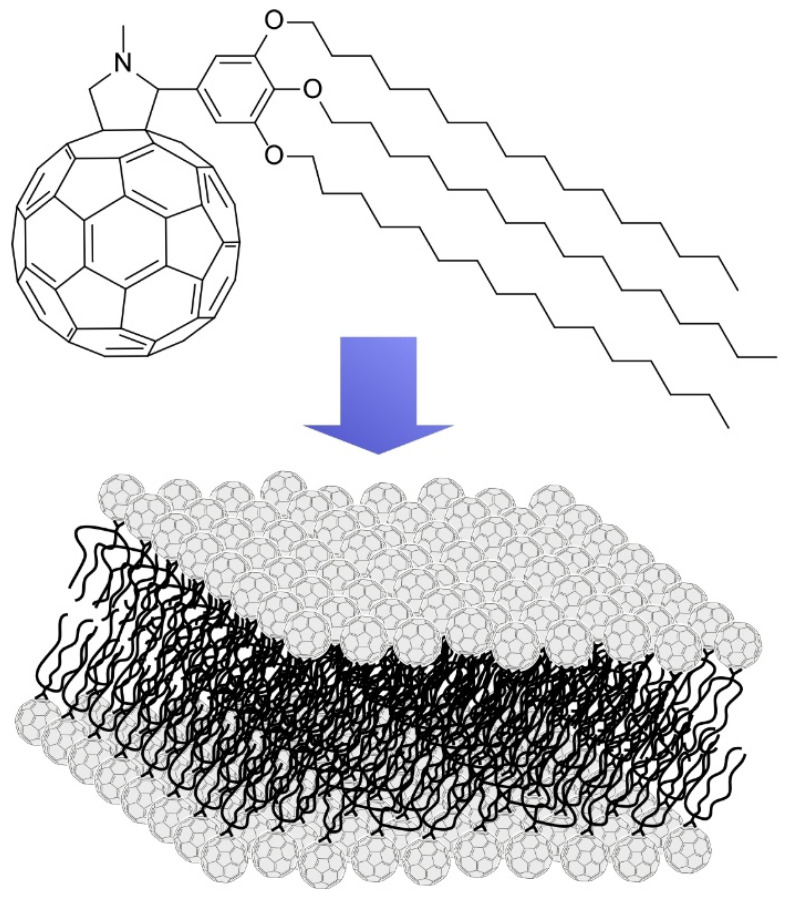
Formation of two-dimensional nanosheet from alkylated C_60_ derivatives with *sp*^3^-carbon-based three C_16_ alkyl chains and *sp*^2^-carbon-based C_60_ moiety.

Nakanishi et al. used alkylated C_60_ derivatives for the fabrication of variously shaped fullerene assemblies (Figure 9) [120]. The used C_60_ derivative possesses *sp*^3^-carbon-based three C_16_ alkyl chains and *sp*^2^-carbon-based C_60_ moiety in one molecule. Because these molecular parts have different affinities to aliphatic and aromatic solvents, this molecule behaves as a solvent-dependent amphiphile. Accordingly, cast films of this C_60_ derivative from various solvents included variously shaped objects—such as spherical vesicles, one-dimensional and two-dimensional fibers, and tapes, two-dimensional discs, and three-dimensional cones. Self-assembled single bilayer discs were obtained from the brown-colored supernatant of its 1,4-dioxane solution. The discs have uniform two-dimensional motifs with a thickness of 4.4 nm, which corresponds to the thickness of interdigitated bilayer. The two-dimensional bilayer is probably formed through *π*-stacking of C_60_ parts and interdigitation of long alkyl chains.

**Figure 10:**
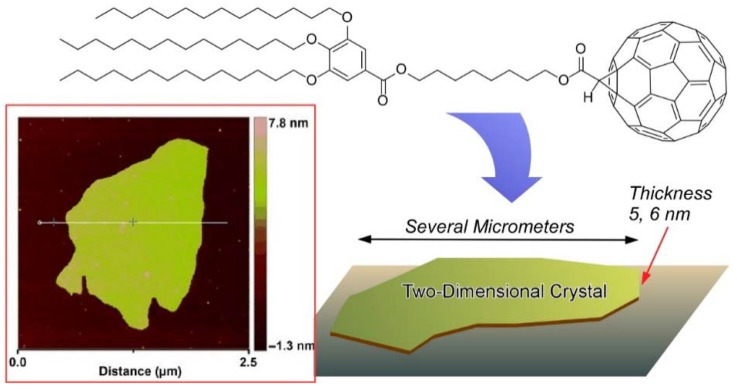
Formation of a thermotropic C_60_ supramolecular nanosheet from molecules consists of a rigid C_60_, a gallic ester segment substituted with three long alkyl chains as the soft part, and a multi-methylene unit as a flexible spacer connecting the two segments. Reprinted with permission from [121]. Copyright 2015 Wiley-VCH.

Tu, Zhu, and coworkers demonstrated a new strategy to introduce a soft group onto fullerene so that the molecules will self-organize to form supramolecular assembly (Figure 10) [121]. They succeeded in the synthesis of a thermotropic C_60_ supramolecular nanosheet with hierarchical structures. A typical molecule consists of a rigid C_60_, a gallic ester segment substituted with three long alkyl chains as the soft part, and a multi-methylene unit as a flexible spacer connecting the two segments. The C_60_ dyads underwent self-organization driven by *π*–*π* interactions to form triple-layer two-dimensional fullerene crystals sandwiched between layers of alkyl chains.

**Figure 11:**
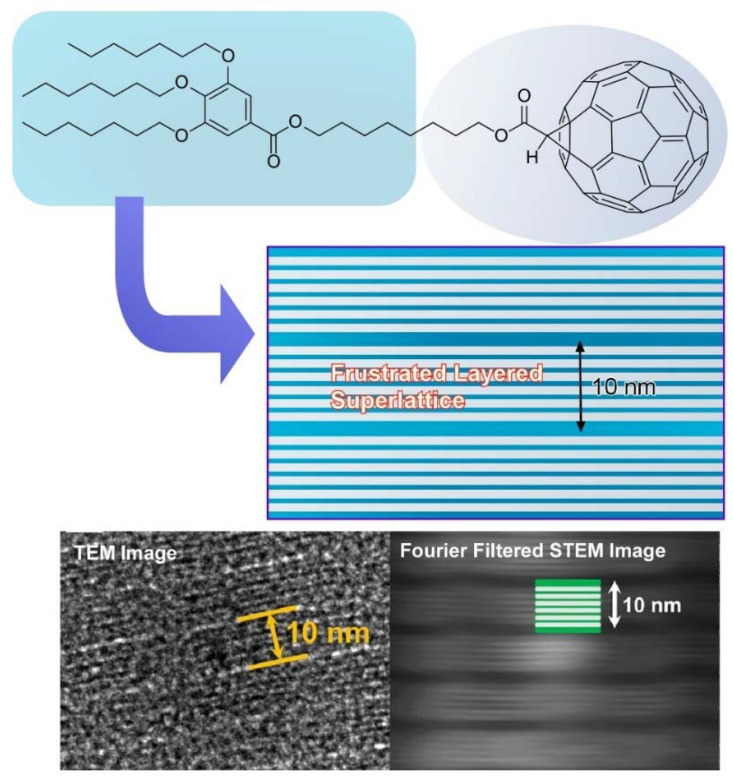
Frustrated layered superlattice as flat nanosheets with sublayer (~2 nm) and superlayer (~10 nm) lamellar thickness. Reprinted with permission from [122]. Copyright 2020 American Chemical Society.

According to the work of the above-mentioned work, Tu, Ungar, and coworkers further synthesized the fullerene block molecule C_7_-C_8_-C_60_ with a rigid fullerene sphere and a soft cone connected by a flexible spacer. They proposed an unprecedented type of self-assembly, where the block molecules organize into a semiconducting superlattice of isolated two-dimensional-ordered C_60_ layers (Figure 11) [122]. The frustration caused by cross-sectional area mismatch between the spheres can be resolved by direction-flipping steric dipoles. The obtained flat nanosheets possess both sublayer (~2 nm) and superlayer (~10 nm) lamellar thickness. Moreover, the superlattice is found to have unexpectedly high electrical conductivity.

## 5. Two-Dimensional Hierarchical Fullerene Nanoarchitecture from Fullerene

Fabrication methods of two-dimensional structures from zero-dimensional fullerene molecules are not limited to direct zero-to-two dimensional nanoarchitectonics. A multi-phase strategy such as zero-to-one to two nanoarchitectonics is also possible. In these cases, hierarchical two-dimensional structures are often obtainable. Minami et al. proposed the fabrication of two-dimensional films of one-dimensional fullerene (C_60_) nanowhiskers that were used as a scaffold for living cells [123]. In the initial step, zero-dimensional C_60_ molecules were converted into one-dimensional fullerene nanowhiskers through the liquid–liquid interfacial precipitation method using toluene as a good solvent and isopropyl alcohol as a poor solvent. The obtained one-dimensional fullerene nanowhiskers were then assembled and aligned at the air–water interface and transferred as a two-dimensional film onto a solid substrate through the conventional LB method. This nanoarchitectonics method provides hierarchical structures of two-dimensional structures in a centimeter area with highly aligned one-dimensional fullerene nanowhiskers. The resulting structures were subjected to cell cultures to control cellular orientation and differentiation to muscle cells. Because of the simplicity and low-cost nature of the proposed approach, this hierarchical nanoarchitectonics strategy would be a promising cell scaffold for tissue engineering.

Krishnan et al. demonstrated a modified method to modulate aligning curvature of one-dimensional fullerene (C_60_) nanowhiskers within the two-dimensional film. Instead of using a conventional LB technique, a newly developed vortex LB method was employed for this purpose [124]. The C_60_ nanowhiskers floated on a water surface were spontaneously assembled according to rotational flows of water subphase. Their alignment curvatures can be controlled at the position from a center of rotating motions. The aligned fullerene nanowhiskers with controlled alignment curvatures were then transferred onto glass substrates used for the culture of bone-forming human osteoblast MG63 cells. It was observed that the growth of the MG63 cells was highly oriented with an axis of aligned one-dimensional C_60_ nanowhiskers. Cell proliferation experiments confirmed the low toxicity nature of the used hierarchical two-dimensional fullerene scaffold, suggesting the potential availability of the proposed method in various biomedical applications.

**Figure 12:**
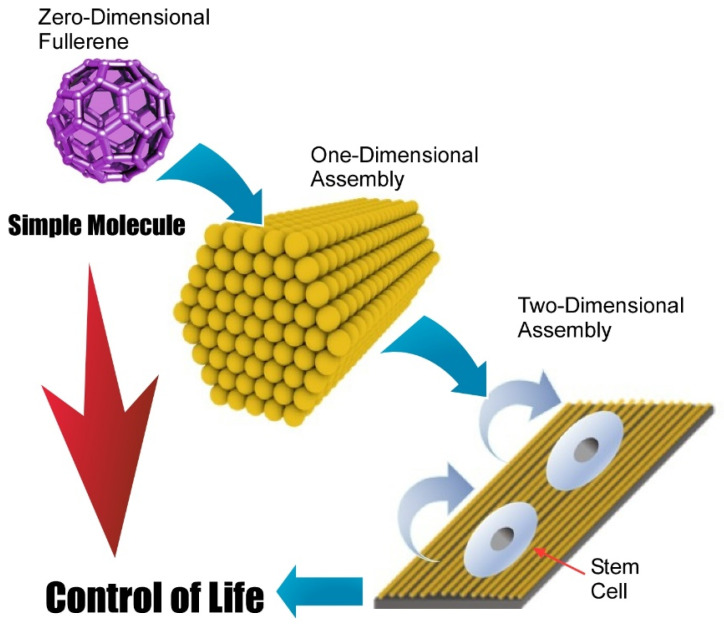
Two-dimensional fullerene (C_60_) nanowhisker nanopatterns with controlled alignments for the culture of human mesenchymal stem.

Very recently, Song et al. investigated the regulation of adhesion and function of human mesenchymal stem cells on two-dimensional fullerene (C_60_) nanowhisker nanopatterns with controlled alignments that were prepared with conventional LB technique (Figure 12) [125]. It was found that regenerative capacity and long-term multipotency were enhanced on two-dimensional nanopatterns with highly aligned fullerene nanowhisker arrays. Appropriately architected nanotopographic surface with certain hydrophobicity gave limited contact area between material surface and cells. This situation induced decreased cell spreading and focal adhesion areas of human mesenchymal stem cells. Enhancement of stemness retention of human mesenchymal stem cells would be related to the diminished cytoskeletal tensions. Regulated contact with surfaces resulted in the appropriate cell contractility with localization of Yes-associated proteins in the nucleus. The proposed strategy would be useful in biomedical applications, such as tissue engineering as a platform for in vitro stem cell expansion. This example demonstrates important roles of two-dimensional assembly to bridge single molecules and life control.

## 6. Future Perspective

In this review, various types of fabrication methods of two-dimensional structures from zero-dimensional fullerene molecules are briefly introduced, which is called ‘zero-to-two nanoarchitectonics’. Although two-dimensional nanomaterials have been paid much attention, most of them were fabricated through material-based physical methods such as exfoliation and chemical vapor depositions. Unlike these common methods, two-dimensional materials described in this review are prepared by supramolecular assembling processes under wet conditions. The presented nanoarchitectonics approaches can be applied to various kinds of solvent-soluble chemical species. In fact, in addition to pristine fullerene molecules, their derivatives and composites are used in the fabrication of two-dimensional materials at interfaces. The presented approaches demonstrated fabrications of various two-dimensional structures including size-tunable hexagonal fullerene nanosheets, two-dimensional fullerene nano-meshes, van der Waals two-dimensional fullerene solids, fullerene/ferrocene hybrid hexagonal nanosheets, fullerene/cobalt porphyrin hybrid nanosheets, two-dimensional fullerene arrays in the supramolecular template, two-dimensional van der Waals supramolecular frameworks, supramolecular fullerene liquid crystals, frustrated layered self-assembly from two-dimensional nanosheets, and hierarchical zero-to-one-to-two fullerene assemblies for cell culture.

Examples shown in this short review article strikingly demonstrated wide varieties in preparation of functional two-dimensional materials even from zero-dimensional nanounits, fullerenes. Therefore, the use of various nano units and their mixtures would create huge possibilities to produce variously functionalized two-dimensional materials, which cannot be obtained from conventional processes such as exfoliation. Coupling with the other interfacial techniques, such as interfacial fabrications of metal–organic frameworks (MOFs) [126] and covalent organic frameworks (COFs) [127], lead to further varieties in structures and functions. Introduction of forefront concepts such as interfacial controls of molecular machines and molecular receptors [128] results in innovative functions in two-dimensional materials. Preparation of two-dimensional materials by bottom-up nanoarchitectonics at interfacial media with novel materials and methodologies would also open new ways in the development of functional materials in various necessary fields such as energy, environmental, and biomedical applications.

## Sample Availability

Samples of the compounds are not available from the authors.
